# P-2282. Infectious Complications Following Lung Transplant for COVID-19 Related Respiratory Failure

**DOI:** 10.1093/ofid/ofae631.2435

**Published:** 2025-01-29

**Authors:** Nandita Kapur, Navina K Birk, Rachna Jayaprakash, Basmah Khalil, Rajan Singh, Zachary W Hanna, Mayur Ramesh, George J Alangaden

**Affiliations:** Henry Ford Hospital, Detroit, Michigan; Henry Ford Hospital, Detroit, Michigan; Henry Ford Hospital, Detroit, Michigan; Henry Ford Hospital, Detroit, Michigan; Henry Ford Hospital, Detroit, Michigan; Henry Ford Health, Detroit, MI; Henry Ford Hospital, Detroit, Michigan; Henry Ford Health, Detroit, MI

## Abstract

**Background:**

Lung transplantation (LT) is a potentially life-saving treatment option for COVID-19 related irreversible respiratory failure. Early outcomes for 1-year survival and graft failure rates among LT recipients (LTr) for COVID-19 related respiratory failure is similar to LTr for non-COVID etiologies. However, the infectious, real-world analysis of complications following LT among LTr for COVID-19 related respiratory failure in comparison to LTr in COVID-19 unrelated respiratory failure has not been described. Our objective was to compare the post-LT infectious complications among LTr for COVID-19 related and unrelated respiratory failure.

**Figure:**
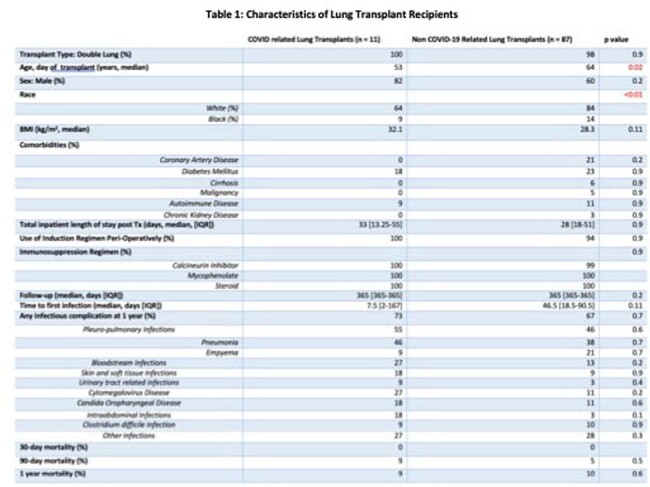

**Methods:**

We analyzed all consecutive LT done at Henry Ford Health System from January 2020 to October 2023. All patients received standard antimicrobial prophylaxis. Demographic data was obtained. The primary outcome was the rate of any infectious complication within one year from transplantation. Secondary outcomes were rates of specific infections, time to infection, and all-cause mortality at 30 days, 90 days, and 1- year.

**Results:**

A total of 98 lung transplantations were done at our center from January 2020 to October 2023. COVID-19 related LTr accounted for 11% of transplants. Median time of follow up was 365 days (187-365). COVID-19 related LTr were younger (median 53 vs. 64 years [p 0.02]) and predominantly white race (p < 0.01). Comorbidities amongst both groups were similar. Rates of post-LTr infectious complications were similar among the two groups, 66% overall. Time to first infection was shorter in the COVID-19 related LTr cohort, however this did not reach significance. Pleuropulmonary infections predominated overall (47%). All-cause post-LT mortality was similar in both groups (Table 1).

**Conclusion:**

Patients receiving LT for COVID-19 related respiratory failure have similar, high rates of infectious complications compared to patients with non-COVID-19 related etiologies, but mortality remains low. LT for COVID-19 related respiratory failure is an acceptable modality of treatment.

**Disclosures:**

Mayur Ramesh, MD, AstraZeneca: Advisor/Consultant|Moderna: Advisor/Consultant|Pfizer: Advisor/Consultant

